# *In Vitro* Evaluation of the Virulence Attributes of Oropharyngeal *Candida* Species Isolated from People Living with Human Immunodeficiency Virus with Oropharyngeal Candidiasis on Antiretroviral Therapy

**DOI:** 10.21203/rs.3.rs-4371952/v1

**Published:** 2024-05-07

**Authors:** Benson Musinguzi, Andrew Akampurira, Hope Derick, Alex Mwesigwa, Edson Mwebesa, Vicent Mwesigye, Immaculate Kabajulizi, Tahalu Sekulima, Francis Ocheng, Herbert Itabangi, Gerald Mboowa, Obondo James Sande, Beatrice Achan

**Affiliations:** Department of Medical Laboratory Sciences, Faculty of Health Sciences, Muni University, P. O. Box 725, Arua, Uganda.; Department of Medical Microbiology, School of Biomedical Sciences, College of Health Sciences, P.O. Box 7072, Makerere University, Kampala, Uganda; Department of Medical Laboratory Sciences, Faculty of Health Sciences, Muni University, P. O. Box 725, Arua, Uganda; Department of Microbiology and Immunology, School of Medicine, P.O Box 317, Kabale University Kabale, Uganda; Department of Mathematics, Faculty of Science, Muni University, P. O. Box 725, Arua, Uganda; Department of Medical Laboratory Sciences, Faculty of Medicine, Mbarara University of Science and Technology, P.O. Box 1410, Mbarara, Uganda; Mycology Unit, Department of Microbiology, Faculty of Medicine, Mbarara University of Science and Technology, P.O. Box 1410, Mbarara, Uganda; Department of Biotechnical and Diagnostic Sciences, College of Veterinary Medicine, Animal Resources and Biosecurity, Makerere University, P.O. Box 7072, Kampala, Uganda.; Department of Dentistry, School of Health Sciences, College of Health Sciences, Makerere University, P.O. Box 7072, Kampala, Uganda.; Department of Microbiology and Immunology, Faculty of Health Sciences, Busitema University, P.O Box 1460, Mbale, Uganda.; African Centre of Excellence in Bioinformatics and Data-Intensive Sciences, Infectious Diseases Institute, College of Health Sciences, Makerere University, P.O Box 22418, Kampala, Uganda; Department of Immunology and Molecular Biology, School of Biomedical Sciences, College of Health Sciences, Makerere University, P.O. Box 7072, Kampala, Uganda.; Department of Medical Microbiology, School of Biomedical Sciences, College of Health Sciences, P.O. Box 7072, Makerere University, Kampala, Uganda.

**Keywords:** Oropharyngeal candidiasis, Candida species, phospholipase, proteinase, virulence

## Abstract

**Background:**

Oropharyngeal *Candida* species are part commensal microflora in the the oral cavity of health individuals. Commensal *Candida* species can become opportunist and transition to pathogenic causes of oropharyngeal candidiasis (OPC) in individuals with impaired immunity through ecological cues and expression of virulence factors. Limited studies have evaluated virulence attributes of oropharyngeal *Candida* species among people living with human immunodeficiency virus (PLHIV) with OPC on antiretroviral therapy (ART) in Uganda.

**Objective:**

Evaluation of the Virulence Attributes of Oropharyngeal *Candida* Species Isolated from People Living with Human Immunodeficiency Virus with Oropharyngeal Candidiasis on Antiretroviral Therapy

**Methods:**

Thirty-five (35) *Candida* isolates from PLHIV with OPC on ART were retrieved from sample repository and evaluated for phospholipase activity using the egg yolk agar method, proteinase activity using the bovine serum albumin agar method, hemolysin activity using the blood agar plate method, esterase activity using the Tween 80 opacity test medium method, coagulase activity using the classical tube method and biofilm formation using the microtiter plate assay method *in vitro*.

**Results:**

Phospholipase and proteinase activities were detected in 33/35 (94.3%) and 31/35 (88.6%) of the strains, respectively. Up to 25/35 (71.4%) of the strains exhibited biofilm formation while esterase activity was demonstrated in 23/35 (65.7%) of the strains. Fewer isolates 21/35 (60%) of the strains produced hemolysin and coagulase production was the least virulence activity detected in 18/35 (51.4%).

**Conclusion:**

Phospholipase and proteinase activities were the strongest virulence attributes of oropharyngeal *Candida* species.

## INTRODUCTION

Oropharyngeal *Candida* species have ability able to exist as commensals in the oral cavity of up to approximately 70% of healthy individuals [1], [2]. The epithelial physical barrier, lysozyme, saliva, histidine-rich polypeptides, lactoferrin, cell-mediated immunity and salivary IgA in the oral cavity play vital roles in keeping oropharyngeal *Candida* species nonpathogenic [3]. However, *C. albicans* and non *albicans Candida* species (NAC) have been reported to cause oropharyngeal candidiasis (OPC) among people living with human immunodeficiency virus (PLHIV) whose CD4 T lymphocyte count is less than 200 cells/μL [4], [5]

The transition of *Candida* species from harmless microflora to pathogenic *Candida* depends on imbalances between reduced host immunity and *Candida* ecological clues [4], [5]. Production of extracellular hydrolytic enzymes and biofilm formation play vital role in OPC pathogenesis [6], [7], [8]. For instance, phospholipases disrupt host cell phospholipids, leading to host cell lysis and tissue invasion while proteinases interrupt surface proteins and defense mechanisms, resulting to tissue invasion [9], [10]. Whereas hemolysin lyses host erythrocytes and obtains iron for metabolism, facilitating host tissue invasion, esterase enzymes have the ability to hydrolyze ester bonds, enhancing binding to host cells, penetration and invasion [11]. Additionally, coagulase binds to fibrinogen and activates prothrombin to convert fibrinogen to fibrin, leading to the clotting of plasma (fibrin clot) which protects *Candida* species from being phagocytosed by granulocytes [12].

Biofilm production and maintenance within the biofilm protect *Candida* against the environment, antifungal drugs, host immune defense, and chemical and physical stresses, leading to antifungal treatment failure and the progression of OPC [8]. This has made extracellular hydrolytic enzymes and biofilm production among *Candida* species vital phenotypic markers for differentiating pathogenic strains that potentiate OPC causation from nonpathogenic [13].

In an effort to reduce the incidence of opportunistic infections such as OPC and improve the quality of life among PLHIV, Uganda implemented the Universal Test and Treatment (UTT) policy in 2017, in which PLHIV, regardless of CD4 cell count are initiated on lifelong antiretroviral therapy (ART) [14]. A recent study reported a 7.6% prevalence of OPC caused by both *C. albicans* and non-albicans *Candida* species in the ART era [15]. However, there are limited reports on the virulence of *Candida* species in the setting of immune suppression in individuals with HIV/AIDS on ART in Uganda. Investigating virulence attributes is vital for understanding pathogenesis and management of OPC among PLHIV. Therefore, we evaluated phospholipase, proteinase, hemolysin, and esterase coagulase production and biofilm formation in oropharyngeal *Candida* species isolated from PLHIV with OPC on ART at AIDS Support Organization (TASO) clinics in Uganda.

## MATERIALS AND METHODS

### Study design and setting

This was a cross-sectional study in which 35 oropharyngeal *Candida* isolates obtained from twenty-nine (29) PLHIV with OPC on ART at TASO Mulago National Referral Hospital and TASO Mbarara Regional Referral Hospital in Uganda were retrieved from the sample repository of our previous study titled “Distribution and antifungal susceptibility profile of oropharyngeal *Candida* species isolated from People Living with HIV in the Era of Universal Test and Treatment Policy in Uganda’’.

### Ethical approval

This study was approved by the Makerere University School of Biomedical Sciences Research Ethics Committee (Reference Number; SBS-2022–254). In addition, administrative clearance to conduct the study was obtained from The AIDS Support Organization (TASO) Uganda Limited headquarters (Reference; TASO/ADMCOO3/2023-UG-REC-009). Written informed consent was obtained from the study participants before being enrolled in the study

### Retrieved oropharyngeal *Candida* isolates

A total of 35 *Candida* isolates were retrieved from sample repository, 20 were *C. albicans*, while 15 were NAC. The NAC species comprised of *Candida tropicalis* (n = 4), *C. glabrata* (n = 4), *C. parapsilosis* (n = 2), *C. dubliniensis* (n = 2), *C. krusei* (n = 2), and *C. lusitaniae* (n = 1) previously identified using a matrix-assisted laser desorption ionization time-of-flight mass spectrometry (MALDI-TOF MS) BioTyper 4.1 system (Bruker Daltonics) from the Mycology/Microbiology (College of American Pathologists accredited) laboratory at the Department of Medical Microbiology, College of Health Sciences, Makerere University, Uganda.

### Preparation of retrieved oropharyngeal Candida isolates for virulence studies

*Candida* isolates that were preserved in brain heart infusion (BHI) broth and 10% glycerol at −80°C glycerol and brain heart infusion (BHI) were retrieved from sample repository freezer. The isolates were thawed by gently warming them to room temperature for 1 hour. A loopful of the culture was streaked onto sabouraud dextrose agar (Oxoid, Basingstoke, UK) supplemented with 50μg/1 ml gentamicin and then incubated at 37°C for 24–48 hours to isolate *Candida* species and the strains were suspended in sterile phosphate-buffered saline (PBS) and matched to 0.5 Mc Farland to carry out the virulence assays. For each virulence factor each isolate was tested in duplicate on two different occasions, and the mean of the 2 values was calculated.

### Determination of phospholipase activity

The phospholipase activity was analyzed using the egg yolk agar method [16], [17]. Briefly, the egg yolk medium comprised Sabouraud dextrose agar (45.5g), sodium chloride (20.43g), calcium chloride (0.193 g), 70 ml of 10% v/v egg yolk emulsion and 630 ml of distilled water as previously described by Tsang et al. [18]. Approximately 10 μl of a standardized yeast suspension (10^8^ CFU/ml) was pipetted, spotted onto fresh egg yolk agar plates and left to dry in a biosafety cabinet. The culture was then incubated at 37°C aerobically for 48 hours, after which the diameter of the precipitation zone around the colony was determined. Phospholipase activity (Pz) was measured by dividing the diameter of the colony (Cd) by the total diameter of the colony plus the precipitation zone (Pd). *C. albicans* ATCC 10231 was chosen as a positive control, while *C. kefyr* 2512 was used as a negative control [19].


$$Pz\;\text{value}\;=\frac{Cd}{Cd+Pd}$$

Pz activity was scored as negative when Pz=1, weak when Pz=0.64-0.99, and strong when Pz≤0.63, as previously described by Price *et al*., [17], indicating that the lower the Pz is, the greater the phospholipase activity.

### Determination of proteinase activity

Proteinase activity was determined using the bovine serum albumin agar (BSA) method [20]. The BSA consisted of 0.1% KH2PO4, 0.05% MgSO4, 2% dextrose, 2% agar and 1% bovine serum albumin as previously described by Tsang et al. [18], and its final pH was adjusted to 4.5 using 1 M HCl and 1 M NaOH. Using a 24-hour-old culture, a yeast suspension of approximately 1×10^8^ CFU/ml was prepared using 1 ml of 0.85% normal saline and a turbidometer. Ten microliters of the standardized yeast suspension was pipetted and spotted onto sterile BSA agar plates. The inoculated plates were then incubated at 37°C for 5 days under aerobic conditions. After incubation, the plates were fixed with 20% trichloroacetic acid and stained with 0.25% w/v Coomassie blue. Decolorization was performed by flooding the culture plates with 15% acetic acid. Proteinase activity was detected by the presence of a clear halo around the yeast colonies. The diameter of the halo clearance (Hd) zone relative to the diameter of the colonies (Cd) was used to assess the degree of proteinase activity (Prz) as previously described by Price et al. [17]. *C. albicans* ATCC 10231 was used as a positive *control*, *and C. kefyr* 2512 was used as a negative control for this experiment. $Prz\;value=\frac{Cd}{Cd+Hd}$

Prz activity was scored as negative when the Prz value = 1, weak when Prz=0.64-0.99, and strong when Prz≤0.63, meaning that a low Prz value indicated strong production of the proteinase enzyme [17].

### Determination of hemolysin activity

The hemolytic activity of *Candida* species was determined by the blood agar plate method [21]. Briefly, SDA (Oxoid) containing 7% sheep blood and 3% w/v glucose with the final pH adjusted to 5.6 ± 0.2 was used. Ten (10) microliters of standardized yeast suspension (10^8^) was inoculated onto blood agar plates, which were then incubated at 37°C in 5% carbon dioxide for 48 hours. After incubation, a transparent/semitransparent zone around the inoculation site was considered to indicate positive hemolytic activity [21]. Beta (β), alpha (α) and gamma (γ) hemolysis was indicated by clear, greenish and no hemolysis, respectively. The ratio of the diameter of the colony (Cd) to that of the translucent zone of hemolysis (Hd) (mm) was used as the hemolytic index (Hz value). *C. albicans* ATCC 90028 was used as a positive control, and *C. parapsilosis* ATCC 2201 was used as a negative control.


$$Hz\;\text{value}\;=\frac{Cd}{Hd}$$

### Determination of esterase activity

The esterase activity of *Candida* species was detected using the Tween 80 opacity test medium method [22]. The test medium was adjusted to a pH of 6.8 and consisted of 1% peptone, 0.5% NaCl, 0.01% CaCl_2_ and 1.5% agar. After the medium cooled (to 50°C), 0.5% Tween-80 was added. Ten microliters of the previously prepared suspension was carefully spotted on Tween 80 opacity test medium and left to dry. This mixture was then incubated at 35°C for 2–5 days under aerobic conditions. The presence of a halo around an inoculated site on tween 80 opacity test medium indicated positive esterase activity [23]. Esterase activity (Ez) was determined as the ratio of the diameter of the colonies (Cd) to the diameter of the clear halo with calcium precipitates around the colony (Hd) as previously described by Price *et al*. [17]. *C. albicans* ATCC10231 served as a positive control for this experiment.


$$Ez\;value=\frac{Cd}{Hd}$$

Ez activity is negative when Ez=1, weak when Ez=0.64-0.99 and strong when Ez≤0.63. Thus, a low Ez value indicated strong esterase production.

### Determination of Coagulase Activity

Coagulase activity was determined by the classical tube coagulase method [24]. Briefly, *Candida* isolates were first standardized to match 0.5 McFarland’s turbidity standard, and 0.5 ml of this standardized cell suspension was added to 0.5 ml of 10% rabbit plasma in a tube. The inoculated tubes were then incubated at 37°C and observed for clot formation after 4 hours. The presence of a visible clot that could not be resuspended by gentle shaking was considered coagulase positive. Test tubes without clots were re-incubated at 37°C and re-examined after 24 hours. *Staphylococcus aureus* ATCC 25923 was used as a positive control, while *Staphylococcus epidermidis* ATCC 14990 served as a negative control.

### Determination of biofilm formation

The ability of *Candida* isolates to form biofilms was analyzed using the microtiter plate assay (Mtp) method[25]. Fresh 24-hour-old broth from *Candida* cultures was used for this assay. The cultures were grown in yeast peptone dextrose (YPD) broth, and the cell suspension was then adjusted to McFarland’s standard using fresh YPD broth. This suspension was further diluted 20-fold to a concentration of approximately 5×10^6^ CFU/ml. Then, 180 μl of sterile YPD broth was aseptically transferred to sterile 96-well polystyrene microtiter plates. For each isolate, 3 wells in a column were used to produce triplicate results. Then, 20 μl of the standardized yeast suspensions were added to each well containing 180 μl of YPD for a final concentration of approximately 5×10^5^ CFU/ml. The microtiter plates were then incubated at 37°C for 48 hrs. At the end of the incubation period, excess planktonic cells and broth were removed, and the plates were washed with phosphate-buffered saline 3 times to remove unattached cells and media components. The plates were blotted with blotting paper, inverted, and left to dry. The sessile cells from which biofilms formed were then fixed by adding 150 μl of methanol to the wells for 20 minutes. The methanol was then removed by inverting the plates, and the liquid was removed. The plates were then washed with phosphate-buffered saline and dried off with blotting paper. Two hundred microliters of 1% crystal violet was added, and the mixture was left to stand for 15 min at 37°C. At the end of the incubation period, crystal violet was added, and the plates were washed 3 times with Dulbecco’s phosphate-buffered saline and left to air dry. After air drying, the stained biofilms were resolubilized using 150 μl of 33% glacial acetic acid, and the plates were carefully agitated using a rotatory shaker. The optical density of the microtiter plates was measured spectrophotometrically at 620 nm with a spectrophotometer at 600 nm. *Candida albicans* ATCC 10231 was used as a positive control, while uninoculated wells that contained sterile YPD were used as negative controls and treated as blanks. The blank absorbance values (ODc) were used to determine whether biofilms were formed by the isolates. The wells containing isolates whose optical density (OD) values were greater than that of the blank well were considered biofilm producers.

The OD values were used to calculate the cutoff values (ODc) of the isolates for biofilm formation categorization and interpretation as previously described by Kırmusaoglu *et al*.[25] as follows: OD ≤ ODc = no biofilm production, ODc < OD ≤ 2ODc = weak biofilm production, 2ODc < OD ≤ 4ODc = moderate biofilm production, 4ODc < OD = strong biofilm producer

### Data analysis plan

Statistical analyses were performed using Stata version 17.0 software. Descriptive statistics, proportions and means were used to summarize the distributions of different virulence attributes. The chi-square test was used to test the association between biofilm formation, coagulase activity between *C. albicans* and NAC. Student’s t test was used to determine the mean difference in phospholipase, proteinase, hemolysin and esterase activities between NAC and *C. albicans*, considering a *P* value < 0.05 to indicate statistical significance.

## RESULTS

A total of 35 *Candida* isolates including *C. albicans* (n = 20), and 15 NAC; *Candida tropicalis* (n = 4), *C. glabrata* (n = 4), *C. parapsilosis* (n = 2), *C. dubliniensis* (n = 2), *C. krusei* (n = 2), and *C. lusitaniae* (n = 1) isolated from PLHIV with OPC on ART at the AIDS Support Organization (TASO) clinics in Uganda were evaluated for phospholipase, proteinase, hemolysin, esterase and coagulase production and biofilm formation.

**Phospholipase (Pz) activity** was detected in 33/35 (94.3%) of the total isolates and in 20/20 (100%) and 13/15 (86.7%) of the *C. albicans* and NAC isolates, respectively. In terms of specific NAC species, all 4/4 (100%) *C. glabrata*, 2/2 (100%) *C. parapsilosis*, 4/4 (100%) *C. tropicalis* and 2/2 (100%) *C. krusei* strains and 1/2 (50%) *C. dubliniensis* strains exhibited Pz activity. Furthermore, 14/20 (70%) *C. albicans*, 2/4(50%) C. *tropicalis* and 1/2 (50%) C. *krusei* strains had strong Pz activity (Table 1). Additionally, the Pz values ranged from 0.39 to 0.94 for *C. albicans* and from 0.38 to 0.79 for the NAC isolates, and no significant difference was noted in the mean Pz values of the *C. albicans* (0.58 ± 0.22) and NAC isolates (0.57 ± 0.13; *p* = 0.88; ) (Table 2).

**Proteinase (Prz) activity** was noted in 31/35 (88.6%) of the total isolates and in 19/20 (95%) and 12/15 (80%) of the *C. albicans* and NAC isolates, respectively. All 4/4 (100%) *C. glabrata*, 4/4 (100%) *C tropicalis*, 2/2 (100%) *C*. krusei, 1/2 (50%) *C. dubliniensis* and 1/2 (50%) *C. parapsilosis* isolates exhibited Prz activity (Table 1). Generally, 13/20 (65%) *C. albicans*, 1/2(50%) *C. dubliniensis*, and 2/4 (50%) *C. glabrata* strains had strong Prz activity. Furthermore, the Prz values ranged from 0.43 to 0.92 for the *C. albicans* isolates and from 0.4 to 0.83 for the NAC isolates. However, there was no significant difference in the mean Prz values of the *C. albicans* (0.65 ± 0.19) and NAC isolates (0.59 ± 0.16; *p* = 0.37) (Table 2)

**Hemolytic (Hz) activity** was noted in 21/35 (60%) of the total isolates, 12/20 (60%) of the *C. albicans* isolates and 9/15 (60%) of the NAC isolates. All 4/4 (100%) C. glabrata, 2/2 (100%) *C. krusei*, and 3/4 (75%) of the *C tropicalis* isolates exhibited Hz activity. Additionally, 10/20 (50%) *C. albicans* and 1/4(25%) *C tropicalis* and strains had strong Hz activity (Table 1). The Hz values ranged from 0.43 to 0.93 for the *C. albicans* isolates and from 0.46 to 0.93 for the NAC isolates. No significant difference was noted in the mean Hz values of the *C. albicans* (0.72 ± 0.2) and NAC isolates (0.68 ± 0.19; *p* = 0.65) (Table 2).

**Esterase (Ez) activity** was detected in 23/35 (65.7%) of the total isolates, 16/20 (80%) of the *C. albicans* isolates and 7/15 (46.7%) of the NAC isolates. Furthermore, 3/4 (75%) of the *C. glabrata* isolates, 3/4 (75%) of the *C tropicalis isolates*, and 1/2 (50%) of the *C. krusei* isolates exhibited Ez activity. Furthermore, 1/2 (50%) *C. krusei*, 7/20 (35%) *C. albicans* and 1/4(25%) *C tropicalis* and 1/4 (25%) *C. glabrata* strains had strong Ez activity (Table 1). The Ez values ranged from 0.35 to 0.83 for the *C. albicans* isolates and from 0.56 to 0.93 for the NAC isolates. Significant difference was noted in the mean Ez values of the *C. albicans* (0.57 ± 0.17) and NAC isolates (0.70 ± 0.14; p = 0.04) (Table 2).

**Coagulase activity** was observed in 18/35 (51.4%) of the *Candida* isolates and in 13/20 (65%) and 5/15 (33.3%) of the *C. albicans* and NAC isolates, respectively. Among the specific NAC isolates, 1/2 (50%) of the *C. dubiniensis* isolates, 1/2 (50%) of the *C. glabrata* isolates and 1/2 (50%) of the *C tropicalis* isolates had coagulase activity while all *C. lusitaniae*, *C. parapsilosis* and *C. krusei* had no coagulase activity (Table 1).

**Biofilm formation** was noted in 25/35 (71.4%) of the total isolates and in 13/20 (65%) and 12/15 (80%) of the *C. albicans* and NAC isolates, respectively. Additionally, 4/4 (100%) *C. glabrata*, 1/1 (100%) C. lusitaniae, 1/2 (50%) *C. dubliniensis*, 1/2 (50%) *C. parapsilosis*, 2/4 (50%) *C tropicalis* and 1/2 (50%) *C. krusei* isolates were biofilm producers. Furthermore, 1/1 (100%) *C. lusitaniae*, 2/4(50) *C. glabrata*, 1/2 (50%) *C. parapsilosis*, 9/20 (45%) *C. albicans*, 7/20 (35%) *C. albicans* strains were strong biofilm producers (Table 1).

## DISCUSSION

The virulence of oropharyngeal *Candida* species, such as the production of extracellular hydrolytic enzymes and biofilm formation, plays a vital role in the pathogenesis of OPC among PLHIV [9], [26]. In this study, we evaluated phospholipase, proteinase, hemolysin,esterase, coagulase activity and biofilm formation by oropharyngeal *C. albicans* and NAC species.

Phospholipases disrupt host cell phospholipids, leading to cell lysis [9], [10]. In our study, phospholipase activity was observed in 94.3% of the total isolates, 100% of the *C. albicans* isolates and 86.7% of the NAC isolates. These results are consistent with other studies that reported that 100% of *C. albicans* had phospholipase activity compared to the low activity of NAC [27], [28]. In contrast to our study, Gokce et al. [29] reported that 60.3% of *C. albicans* isolates demonstrated phospholipase activity, and all NAC isolates were phospholipase negative. It has been demonstrated that stronger phospholipase activity in oral *C. albicans* is due to its germ tube induction potential, which facilitates mucosa penetration [30]. Additionally, reports have shown that *C. albicans* has increased levels of phospholipase activity, which allows it to acquire nutrients from the host and contributes to host invasion [9], [31], [32].

Proteinases disrupt host surface proteins and defense mechanisms, leading to tissue invasion [9], [10]. Proteinase activity was noted in 95% of the *C. albicans* isolates and 80% of the NAC isolates in this study. This finding is in agreement with previous studies by Deepa et al. [33], who observed proteinase activity in 90% of *C. albicans* and 81.3% of NAC isolates; and Mane et al. [19], who noted proteinase activity in 87.8% of *C. albicans* isolates. All *C. glabrata*, *C tropicalis* and *C. krusei* isolates were proteinase producers, which is in agreement with the findings of Deepa et al. [33], who reported that all *C. glabrata* and *C tropicalis* isolates were proteinase producers. A lower pH, xerostomia, and changes in the salivary composition of PLHIV are conducive to proteinase production [34], which could be the reason for the stronger proteinase activity.

Hemolytic activity is caused by the enzyme hemolysin, which lyses host erythrocytes and obtains iron for metabolism, facilitating host tissue invasion. We observed hemolytic activity in 60% of *C. albicans* strains and 100% of *C. glabrata* and *C. krusei strains*. This is in agreement with a study by Fatahinia et al. [22], which reported that 100% of *C. glabrata* and C. *krusei* were hemolysin producers. However, our findings are contrary to studies that have reported that 100% of *C. albicans* strains have hemolytic activity [18], [35], [36], [37]. Additionally, Mane et al. [38] reported that 100% of *C. albicans*, *C. glabrata* and *C. krusei* were hemolysin producers. This may be because *C. albicans* and NAC have the same mechanism of hemolytic activity by secreting the hemolysin enzyme that lyses host erythrocytes and obtains iron for their own metabolism and survival, facilitating host tissue invasion. In addition, candidalysin toxin contribute to red blood cell lysis in *C. albicans* infections [19], [35].

Esterase enzymes have the ability to hydrolyze ester bonds in the host cell, enhancing binding to the host cell, penetration and invasion [11]. In the present study, esterase activity was detected in 80% of the *C. albicans* isolates and 46.7% of the NAC isolates. This is in agreement with previous studies that demonstrated stronger esterase activity in *C. albicans* than in NAC [39], [40]. Furthermore, approximately 75% of *C. glabrata*, *C. tropicalis*, and 50% of *C. krusei* had esterase activity in this study, which is in agreement with the findings of Deepa et al. [33], who reported esterase activity in these same NAC isolates. In contrast to our results, Pandey et al. [9] reported that 56.3% of *C. albicans* had esterase activity but no esterase activity in *C. glabrata* and *C. krusei*, while Fatahinia et al. [22] reported esterase activity in 100% of *C. albicans*, *C. glabrata*, *C. krusei* and *C. dubliniensis*. The expression of enzymes by different *Candida* species may vary based on the *Candida* ecological clues, stage of infection and host immune response [39], [40], which could be the reason for the variance in different studies.

Coagulase binds to fibrinogen and activates prothrombin to convert fibrinogen to fibrin, leading to the clotting of plasma which protects *Candida* species from being phagocytosed by granulocytes [12]. Coagulase activity was detected in 51.4% of the *Candida* isolates, 65% of the *C. albicans* isolates and 33.3% of the NAC isolates. Approximately 50% of the *C. dubiniensis*, *C. glabrata* and *C tropicalis* isolates exhibited coagulase activity in this study. This result is consistent with a study by Yigit et al. [24], reporting coagulant activity in *C. albicans* (64.7%). However, our findings disagree with those of Rodrigues et al. [41], who reported strong activity in *C. abicans* (88%), and Gupta et al. [42], who reported low coagulase activity in both *C. abicans* (25.9%) and *C. tropicalis* (28.8%). The variability in coagulase activity among different studies and species could be due to differences in the pathogenicity of different *Candida* species and host immune status and differences in coagulase activity detection techniques, as we used rabbit plasma since it has been indicated that rabbit plasma has greater sensitivity to coagulase activity in *Candida* species than sheep and human plasma [24]

Biofilm formation is defined as structured microbial communities adhered to a surface and enclosed in a matrix of exopolymeric material [43]. The biofilm produced by *Candida* species and remaining within the oral cavity biofilm protects it against the environment, antifungal drugs, host immune defense, chemical and physical stresses, leading to antifungal treatment failure and the incidence of OPC [8]. Biofilm formation was noted in 71.4% of *Candida* isolates and was more common in the NAC (80%) than in the *C. candida* (65%) isolates. All *C. glabrata*, *C. lusitaniae*, *C. parapsilosis*, and *C. krusei* isolates and 75% of *C tropicalis were* biofilm producers. Our results are consistent with other studies that have observed slightly greater biofilm production in NAC than in *C. albicans isolates* [44], [45]. The greater biofilm formation of NAC species than that of *C. albicans* could be due to differences in cell surface adhesion mechanisms, cell morphology and gene expression [46], [47]. These differences may increase the ability of NAC species to adhere to epithelial cells, develop antifungal resistance and evade host immune responses compared to those of *C. albicans* [46].

### Limitations

We acknowledge that we may have been underpowered to comprehensively compare virulence attributes among *C. albicans* and NAC species, as the sample size of the NAC species was small. Additionally, we did not have HIV negative and ART naïve control group.

## CONCLUSION

Phospholipase and proteinase activities were the strongest virulence attributes of oropharyngeal *Candida* species. *C. albicans* exhibited increased extracellular hydrolytic enzyme activities as compared to NAC species.

## Figures and Tables

**Table 1:** Extracellular hydrolytic enzymatic activity and biofilm formation by different Candida species

Virulence	Score	Activity index	C. albicans (20) n (%)	Non albicans Candida (15) Total (35)						
				C. dubliniensis	C. glabrata	C. lusitaniae	C. parapsilosis	C. tropicalis	C. krusei	n (%)
				(2) n (%)	(4) n (%)	(1) n (%)	(2) n (%)	(4) n (%)	(2) n (%)	
Phospholipase	Strong	≤ 0.63	14(70)	0(00)	1(25)	0(00)	0(00)	2(50)	1(50)	18(51.4)
	Weak	0.64–0.99	6(30)	1(50)	3(75)	0(00)	2(100)	2(50)	1(50)	15(42.9)
	Negative	1	0(0)	1(50)	0(00)	1(100)	0(00)	0(00)	0(00)	2(5.7)
	Strong	≤ 0.63	13(65)	1(50)	2(50)	0(00)	0(00)	1(25)	0(00)	17(48.6)
Proteinase	Weak	0.64–0.99	6(30)	0(00)	2(50)	0(00)	1(50)	3(75)	2(100)	14(40.0)
	Negative	1	1(5)	1(50)	0(00)	1(100)	1(50)	0(00)	0(00)	4(11.4)
	Strong	≤ 0.63	10(50)	0(00)	0(00)	0(00)	0(00)	1(25)	0(00)	11(31.4)
Hemolysin	Weak	0.64–0.99	2(10)	0(00)	4(100)	0(00)	0(00)	2(50)	2(100)	10(28.6)
	Negative	1	8(40)	2(100)	0(00)	1(100)	2(100)	1(25)	0(00)	14(40.0)
	Strong	≤ 0.63	7(35)	0(00)	1(25)	0(00)	0(00)	1(25)	1(50)	10(28.6)
Esterase	Weak	0.64–0.99	9(45)	0(00)	2(50)	0(00)	0(00)	2(50)	0(00)	13(37.1)
	Negative	1	4(20)	2(100)	1(25)	1(100)	2(100)	1(25)	1(50)	12(34.3)
Coagulase	Positive		13(65)	1(50)	2(50)	0(00)	0(00)	2(50)	0(00)	18(51.4)
	Negative		7(35)	1(50)	2(50)	1(100)	2(100)	2(50)	2(100)	17(48.6)
	Strong		9(45)	0(00)	2(50)	1(100)	1(50)	0(00)	0(00)	13(37.1)
Biofilm	Moderate		3(15)	0(00)	1(25)	0(00)	0(00)	2(50)	2(100)	8(22.9)
	Weak		1(05)	0(00)	1(25)	0(00)	1(50)	1(25)	0(00)	4(11.4)
	Negative		7(35)	2(100)	0(00)	0(00)	0(00)	1(25)	0(00)	10(28.6)

n (%) shows the number (n) and percentage (%) of each Candida species exhibiting each virulence attribute.

**Table 2:** Comparison of different extracellular hydrolytic enzymatic activity values of C. albicans and NAC

Candida isolates (*n*)	Phospholipase activity				Proteinase activity				Hemolytic Activity				Esterase activity			
	N (%)	mean ± SD	Range	95% CI	N (%)	mean ± SD	Range	95% CI	N (%)	mean ± SD	Range	95% CI	N (%)	mean ± SD	Range	95% CI
C. albicans (20)	20 (100)	0.58 ± 0.22	0.39–0.94	0.48–0.68	19 (95)	0.65 ± 0.19	0.43–0.92	0.56–0.74	12 (60)	0.72 ± 0.20	0.43–0.93	0.59–0.85	16 (80)	0.57 ± 0.17	0.35–0.83	0.48–0.66
NAC (15)	13 (86.7)	0.57 ± 0.13	0.38–079	0.49–0.65	12 (80)	0.59 ± 0.16	0.4–083	0.49–0.69	9 (60)	0.68 ± 0.19	0.46–0.93	0.53–0.83	7 (46.7)	0.70 ± 0.14	0.56–0.93	0.57–0.83
P value	0.09	0.88			0.17	0.37			1	0.65			0.04	0.04		
Total (35)	33 (94.3)	0.48 ± 0.19	0.37–0.94	0.51–0.64	31 (88.6)	0.63 ± 0.19		0.56–0.69	21 (60)	0.71 ± 0.19	0.43– 093	0.62–0.79	23 (65.7)	0.61 ± 0.17	0.35–0.93	0.54–0.68

The n (%) shows the number (n) and percentage (%) of C. albicans and NAC isolates exhibiting each virulence attribute. The means ± SDs show the mean value and standard deviation of each virulence attribute at 95% confidence interval.

## Data Availability

The analyzed datasets are available from the corresponding author upon reasonable request.
